# Effects of environmental phenols on eGFR: machine learning modeling methods applied to cross-sectional studies

**DOI:** 10.3389/fpubh.2024.1405533

**Published:** 2024-08-01

**Authors:** Lei Liu, Hao Zhou, Xueli Wang, Fukang Wen, Guibin Zhang, Jinao Yu, Hui Shen, Rongrong Huang

**Affiliations:** ^1^Department of Pathology, Affiliated Hospital of Nantong University, Nantong, China; ^2^Department of Thoracic Surgery, Affiliated Hospital of Nantong University, Nantong, China; ^3^Department of Pathology, Qingdao Eighth People’s Hospital, Qingdao, China; ^4^Institute of Computer Science and Engineering, Sun Yat-Sen University, Guangzhou, China; ^5^College of Electronic and Information Engineering, Tongji University, Shanghai, China; ^6^Institute of Computer Science and Engineering, University of Wisconsin-Madison, Madison, WI, United States; ^7^Department of Computer Science and Engineering, The Ohio State University, Columbus, OH, United States; ^8^Department of Pharmacy, Affiliated Hospital of Nantong University, Nantong, China

**Keywords:** environmental exposure, phenols, machine learning, glomerular filtration rate, NHANES

## Abstract

**Purpose:**

Limited investigation is available on the correlation between environmental phenols’ exposure and estimated glomerular filtration rate (eGFR). Our target is established a robust and explainable machine learning (ML) model that associates environmental phenols’ exposure with eGFR.

**Methods:**

Our datasets for constructing the associations between environmental phenols’ and eGFR were collected from the National Health and Nutrition Examination Survey (NHANES, 2013–2016). Five ML models were contained and fine-tuned to eGFR regression by phenols’ exposure. Regression evaluation metrics were used to extract the limitation of the models. The most effective model was then utilized for regression, with interpretation of its features carried out using shapley additive explanations (SHAP) and the game theory python package to represent the model’s regression capacity.

**Results:**

The study identified the top-performing random forest (RF) regressor with a mean absolute error of 0.621 and a coefficient of determination of 0.998 among 3,371 participants. Six environmental phenols with eGFR in linear regression models revealed that the concentrations of triclosan (TCS) and bisphenol S (BPS) in urine were positively correlated with eGFR, and the correlation coefficients were *β* = 0.010 (*p* = 0.026) and *β* = 0.007 (*p* = 0.004) respectively. SHAP values indicate that BPS (1.38), bisphenol F (BPF) (0.97), 2,5-dichlorophenol (0.87), TCS (0.78), BP3 (0.60), bisphenol A (BPA) (0.59) and 2,4-dichlorophenol (0.47) in urinary contributed to the model.

**Conclusion:**

The RF model was efficient in identifying a correlation between phenols’ exposure and eGFR among United States NHANES 2013–2016 participants. The findings indicate that BPA, BPF, and BPS are inversely associated with eGFR.

## 1 Introduction

As a common economic stress and public health event, chronic kidney disease (CKD) has a significant impact on global health and has been recognized as a leading public health problem worldwide (1). The estimated glomerular filtration rate (eGFR) reflects the kidney’s ability to filter blood. It has the characteristics of stable results and high reproducibility. It is an important clinical indicator for evaluating human renal function (2, 3). Not only is it of great value in the prevention, diagnosis and treatment of renal function, but eGFR is also related to other functions of the body. European Society of Cardiology states that the prognostic impact of eGFR on heart failure has been well established, decreased eGFR is a better predictor of adverse outcome than decreased left ventricular ejection fraction (4). A large meta-analysis illustrated that the assessment and inclusion of eGFR will provide some support for cardiovascular risk in the general population (5). Furthermore, eGFR were associated with all-cause mortality among US adults with obstructive lung function (6). Therefore, additional studies of eGFR related factors and development of prevention strategies are necessary to ensure optimal health care. Diabetes and hypertension are the prior factors of abnormal eGFR, but other factors, including environmental toxins, also cause abnormal changes in eGFR (7). Yufen and colleagues studied the relationship between serum concentrations of per-and polyfluoroalkyl substances (PFAS) and kidney damage in 1,700 people over 18 years. The results show that PFAS has a combined effect on eGFR. Perfluorooctane sulfonate (PFOS) concentration is negatively correlated with eGFR, while perfluorohexane sulfonate (PFHS) is positively correlated (8).

Phenolic compounds represented by bisphenol A (BPA) and its substitutes are widely detected in various foods, consumer products, human and animal bodies, and are widely found in multi environmental components such as soil, water and air. It is widely distributed in areas with high levels of urbanization and industrialization and is a typical environmental endocrine disruptor (9). Even at low doses, it can stimulate cellular responses and affect body functions (10). In addition to the self-toxicity of phenolic compounds, their transformation metabolites *in vivo* may have more complex endocrine disrupting and toxic effects than their own compounds (11). Some animal studies have suggested that kidney may be adversely affected by phenolic compounds. BPA deregulates autophagy flux and redox protection mechanisms, exacerbating chronic kidney injury (12). Kapil et al. showed that bisphenol S (BPS) exposure significantly disrupted rat kidney tissue structure, changed kidney injury marker levels, and affected kidney metabolic pathways (13). Epidemiological studies have also found that phenolic compounds may have adverse effects on renal function. Kang evaluated the effect of exposure to phthalates and environmental phenols on eGFR in 9008 adults from 2005 to 2016 National Health and Nutrition Examination Survey (NHANES). Moreover, exposure to BPA may be responsible for declined eGFR and increased albumin-to-creatinine ratio (ACR) (14). However, phenolic compounds are a large class of substances, and previous studies have focused on the analysis of BPA. The association between other phenolic compounds and eGFR lacks support from population data.

Most traditional statistical models have certain requirements or assumptions for the data. However, in most cases, people cannot make any assumptions about the distribution of real-world data, and are prone to over-fitting or under-fitting, making them unrepresentative. Machine learning (ML) does not make any assumptions about the data, and the generated results are judged by the cross-validation method, getting rid of the classic statistical process of assuming distribution, model fitting, hypothesis testing, and *p*-value comparison. It has the advantages of good model prediction effect and cross-validation results that are easily understood by practical workers. Currently commonly used “black box” models, such as one-hot coding mlp and random forest (RF), have been widely used to build medical risk prediction models and determine potential determinants (15, 16).

Therefore, to provide new insights into the potential influencing factors of eGFR, this study utilized data from the 2013–2016 NHANES, fitted a ML interpretability model, to explore the relationship between phenolic exposure and eGFR.

## 2 Materials and methods

### 2.1 Study population

NHANES employs a cross-sectional study design and the survey data is collected in two-year cycles, which is then made available to researchers for analysis. The survey data has been instrumental for epidemiological studies and public health policy decision-making, covering a wide range of topics including the prevalence of chronic diseases, food consumption patterns, and environmental exposures (17). A total of 20,146 participants were considered potential study subjects. After excluding participants missing environmental phenols and serum creatinine data required to calculate eGFR, a total of 3,371 adults aged 20 years and older were included in our final analytic model (Supplementary Figure S1). Multiple imputation was performed to impute missing covariate values. To ensure that all the protocols and procedures implemented by the program align with the highest ethical standards, the National Center for Health Statistics’ ethics review board has approved all NHANES protocols. Additionally, the program respects the rights and privacy of all participants, and written informed consent is always obtained before any data collection takes place.

### 2.2 Detection of environmental phenols

The NHANES employs a technique called online solid-phase extraction, which is coupled with high-performance liquid chromatography and tandem mass spectrometry. By using isotopically labeled internal standards, they are able to detect phenols in non-occupationally exposed subjects’ urine with a limit of 0.1–1.7 micrograms per liter (μg/L) in 100 μL of urine. Following NHANES analysis guidelines, Phenolic below the limit of detection (LOD) were expressed using LOD divided by the square root of two (Statistics 2024). Phenolic substances included in the study include BPA, bisphenol F (BPF), BPS, benzophenone-3 (BP3), triclosan (TCS), 2,5-dichlorophenol (2,5-DCP) and 2,4-dichlorophenol (2,4-DCP) in urinary. Phenolic substances concentrations were standardized by urine creatinine were used to adjust for urine dilution.

### 2.3 eGFR calculation

For eGFR calculation, we used the CKD-Epidemiology Collaboration (EPI) equation. The eGFR calculated by the CKD-EPI equation is considered a better diagnostic tool than the modification of diet in renal disease equation for diagnosing and staging CKD (18, 19). Equation as follows: eGFRCKD−EPI (mL/min/1.73 m2) = 141 × min (Scr/κ, 1) α × max (Scr/κ, 1) -1.209 × 0.993Age × 1.018 [if female] × 1.159 [if black], where Scr denotes serum creatinine concentration that were measured by the Jaffe rate methods, κ is 0.9 for men and 0.7 for women, and α is −0.411 for males and − 0.329 for females.

### 2.4 Covariates

We gathered covariate data on research subjects in Demographics, Laboratory, and Questionnaire Data of NHANES. The following covariate data was collected: 1. General characteristics such as age, gender, ethnicity, education level, marital status, family poverty income ratio (PIR), body mass index (BMI), and past-year alcohol consumption; 2. Medical conditions such as diabetes and hypertension based on whether or not they have ever been informed by a doctor or other health expert. To simplify the covariate grouping, we categorized the covariates briefly, and the detailed feature grouping is shown in Table 1, based on previously published relevant literature.

**Table 1:** Demographic and socio-behavioral characteristics and eGFR of the study population.

	*N* (%)	eGFR (mL/min/1.73 m^2^)^a^	*p-*value^b^
Total	3,371 (100)	94.59 (23.62)	
Gender			0.004
Male	1,580 (46.9)	92.91 (22.33)	
Female	1791 (53.1)	96.08 (24.61)	
Age (year)			<0.001
20–39	1,128 (33.5)	112.45 (17.52)	
40–59	1,143 (33.9)	95.77 (17.36)	
60–79	1,100 (32.6)	75.06 (19.29)	
Race/ethnicity			<0.001
Mexican American	513 (15.2)	100.03 (22.58)	
Other Hispanic	377 (11.2)	95.55 (20.60)	
Non-Hispanic White	1,243 (36.9)	88.00 (22.51)	
Non-Hispanic Black	733 (21.7)	99.02 (27.07)	
Other groups	505 (15.0)	98.17 (20.09)	
Educational level			0.225
Below high school	770 (22.8)	93.44 (24.28)	
High school	746 (22.1)	95.33 (24.54)	
Above high school	1855 (55.1)	94.78 (22.95)	
Body mass index			<0.001
<25 kg/m^2^	935 (27.7)	97.66 (23.80)	
>25 to <30 kg/m^2^	1,102 (32.7)	91.97 (23.37)	
>30 kg/m^2^	1,334 (39.6)	94.61 (23.45)	
Poverty: income ratio			0.009
≤1	682 (20.2)	97.61 (25.66)	
>1	2,689 (79.8)	93.83 (23.01)	
Marital status			0.422
Married/living with partner	2020 (59.9)	94.34 (21.76)	
Widowed/divorced, separated/never married	1,351 (40.1)	94.97 (26.15)	
Drinking			0.23
Yes	2,176 (64.6)	94.72 (22.80)	
No	1,195 (35.4)	94.37 (25.05)	
Hypertension			<0.001
Yes	1,266 (37.6)	83.40 (24.32)	
No	2,105 (62.4)	101.33 (20.42)	
Diabetes			<0.001
Yes	459 (13.6)	83.17 (25.76)	
No	2,912 (86.4)	96.40 (22.75)	

^a^Mean (Standard Deviation). ^b^*P*-value was tested by Chi-square test or analysis of variance.

### 2.5 Statistical analysis

In this study, R 4.2.2 software and python software were used to sort out and analyze the data. A two-sided *p*-value <0.05 was considered statistically significant. Different groups of population characteristics are statistically described using absolute numbers and percentages. In addition, the content and differences of log-transformed eGFR between different groups were explored. For the normally distributed variables, differences were compared by two independent sample t-tests or analysis of variance. For skewed variables, geometric mean, the median and interquartile were used to describe. Prior to statistical analysis, we established generalized additive models to assess potential nonlinear relationships between environmental phenol exposure and eGFR. The results show that the effective degrees of freedom (EDF) of most models are equal to or close to 1. Combined with the consideration of nonlinear *p* values and fitting curves, we believe that the association between eGFR and environmental phenols is more likely to be linear. Therefore, we used multiple linear regression to analyze the linear relationship between phenolic concentration and eGFR, in which the concentration was transformed by natural logarithm. The model adjusted for covariates (age, sex, race, BMI, PIR, diabetes, and hypertension) with significant differences in eGFR levels between groups. Analysis is adjusted for the survey design and weighting factors.

Several sensitivity analyses were conducted to examine the robustness of our results: (a) covariates with no significant difference in eGFR levels between groups were also included in the model for adjustment, including marital status, educational level, and alcohol drinking; (b) we also used Quantile-based g calculation (QGC) to test the associations between environmental phenols joint exposures and eGFR and *p*-value less than 0.05 was considered to have a mixture effect.

### 2.6 ML model strategies

We conducted a study to investigate the impact of phenol exposure on eGFR. To accomplish this, we divided our research data into two parts: 80% for training and 20% for testing. We employed five distinct ML models, namely Adaptive Boosting (AdaBoost), Support Vector Machine (SVM), RF, Decision Tree (DT), and K-Nearest Neighbors (KNN), to analyze the data. In general, AdaBoost is highly accurate for training data, but it may sacrifice accuracy with unbalanced datasets and increase computational time (20). SVM, effective for non-linear and high-dimensional data, remains relatively unaffected by the nature of the data (21). RF excels in analyzing high-dimensional data and is robust against noise, but its time complexity escalates with larger datasets (22). DT stands out for its ease of understanding and capacity for visual analysis, but it’s susceptible to overfitting (23). Lastly, KNN is notable for its accuracy, outlier insensitivity, and simplicity, though it also suffers from high time complexity (24). Each model was chosen for its unique characteristics and potential in eGFR regression. We used a set of features that included population baseline characteristics, electronic health records, and the concentration level of environmental phenols to train five ML models from sci-kit learn on United States NHANES datasets. The models were trained using 17 features training datasets. Since the outcome characteristics are continuous variables, we evaluated the ability of the ML model through regression to generalization using the mean average error and the coefficient of determination to achieve the best model fitting effect. This measure indicates how effective the regression is in predicting outcomes to select a better interpretable model, quantifying how well the independent variables in the regression model explain the variation in the dependent variable (25, 26).

During the training phase, we used the designated training sets to fine-tune these five ML models. The testing sets were then utilized to evaluate their effectiveness. We assessed each model’s distinct features to identify the most appropriate one for kidney detection. To achieve this, we have employed three widely-used evaluation metrics: Mean Squared Error (MSE), Mean Absolute Error (MAE), and the coefficient of determination. These metrics, when combined, provide a holistic view of our models’ accuracy and explanatory power. MSE quantifies the average squared difference between predicted and actual values, offering insight into the magnitude of errors. Conversely, MAE measures the average absolute difference, offering a more intuitive interpretation of the average prediction error. The coefficient of determination represents the proportion of variance in the target variable explained by the model, ranging from 0 (no explanatory power) to 1 (perfect prediction). By combining these metrics, we aim to provide a comprehensive evaluation of our models’ performance. Additionally, we applied shapley additive explanations (SHAP) values to elucidate the chosen model, focusing on impact factors associated with eGFR in participants from 2013 to 2016 (27, 28). This method has been widely used in many medical prediction models.

## 3 Results

### 3.1 Characteristics of the study population

Table 1 shows the general demographic characteristics of the 3,371 subjects, evenly distributed across gender, age groups (male (50.6%); 20–39 (33.5%), 40–59 (33.9%), and 60–79 (32.6%)). The eGFR of total population was 94.59 ± 23.62 mL/min/1.73 m2 (mean ± standard error), with high eGFR appeared to be more likely to be female, Mexican American, have a lower BMI and have no underlying medical conditions. However, there were no significant differences in eGFR levels among the various groups of marital status, education level and alcohol consumption.

### 3.2 Phenolic substance concentration in urine

Table 2 presents descriptive statistics of geometric means and geometric standard deviations of eGFR and environmental phenols levels. Among all the subjects, the geometric means of eGFR level was 90.94 ng/mg creatinine, and the medians was 96.22 ng/mg creatinine. The highest and lowest geometric mean values among environmental phenols are BP3 and BPF respectively, with contents of 17.50 ng/mg creatinine and 0.42 ng/mg creatinine.

**Table 2:** Distribution of eGFR and environmental phenols in urine of the general United States population (*n* = 3,371).

Categories	Geometric	Percentile		
	Mean^a^	25th	50th	75th
eGFR	90.94 (90.00, 91.88)	79.83	96.22	111.21
BPA	1.18 (1.14, 1.22)	0.67	1.11	1.96
BPS	0.52 (0.50, 0.54)	0.23	0.47	1.04
BPF	0.42 (0.40, 0.44)	0.16	0.33	0.81
BP3	17.50 (16.28, 18.82)	4.40	13.42	54.47
TCS	7.71 (7.22, 8.24)	1.72	4.63	22.72
2,5-DCP	4.30 (4.00, 4.61)	0.99	2.76	12.77
2,4-DCP	0.72 (0.69, 0.76)	0.30	0.56	1.32

^a^G-Mean (95 %CI). Environmental phenols in urine corrected using urinary creatinine (ng/mg creatinine).

### 3.3 Association between eGFR and environmental phenols

Table 3 summarizes the association of 6 environmental phenols with eGFR in linear regression models adjusted for covariates including general characteristics and medical conditions. We found that the concentrations of TCS and BPS in urine were positively correlated with eGFR, and the correlation coefficients were *β* = 0.010 (*p* = 0.026) and *β* = 0.007 (*p* = 0.004) respectively. No statistically significant associations were found between eGFR levels and other environmental phenols. It should be noted that beta coefficients of predictors were relatively small because we applied log-transformation with base e on all continuous variable concentrations.

**Table 3:** Association of urinary environmental phenols with eGFR (mL/min/1.73 m^2^) in regression model (*n* = 3,371).

Categories	*β* (95%CI)	*P*-value
BPA	−0.007 (−0.015, 0.001)	0.098
BPS	0.009 (0.003, 0.015)	0.008
BPF	−0.004 (−0.014, 0.005)	0.365
BP3	0.004 (−0.001, 0.010)	0.054
TCS	0.007 (0.003, 0.011)	0.004
2,5-DCP	0.001 (−0.004, 0.005)	0.933
2,4-DCP	0.005 (−0.002, 0.013)	0.149

The association was adjusted for gender, age, race/ethnicity, poverty: income ratio, body mass index, hypertension, and diabetes. Creatinine-corrected urinary ambient phenols and eGFR were log-transformed.

In sensitivity analyses, when additionally adjusting for marital status, educational level, and alcohol drinking, the overall results were consistent with our main analysis, although minor changes were observed (Table S1). When QGC was used to explore the association between combined exposure to environmental phenols and eGFR, there was no evidence of a combined exposure effect (*p* value = 0. 069; Supplementary Figure S2).

### 3.4 Testing the ML models’ performance in predicting eGFR

We apply the trained model to the test set and summarize the key evaluation and interpretable metrics in Table 4. The RF model has the best mean absolute error (MAE) performance (MAE: 0.621) which was significantly better compared to the corresponding MAE values in the other 4 models. However, DT (MAE: 1.77), AdaBoost (MAE: 3.60), and KNN (MAE: 8.25) also demonstrated good performance in prediction eGFR. Moreover Table 4 represents the general performance of the models under evaluation. The Coefficient of determination (0.998) of RF showed the best discriminate on ability among all five ML models. SVM (0.784) and KNN (0.799) have comparable performance on coefficient of determination scores. Finally, comprehensive analysis based on the features demonstrates that RF has the highest precision and resilience for predicting eGFR.

**Table 4:** Comparison of model evaluation metrics among five ML models.

Methods	Mean squared error	Mean absolute error	Coefficient of determination
Random forest	0.680	0.621	0.998
KNN	116.516	8.246	0.779
AdaBoost	20.635	3.604	0.961
Decision tree	213.452	11.772	0.605
SVM	122.211	7.663	0.783

KNN, K-Nearest Neighbors; AdaBoost, Adaptive Boosting; SVM, Support Vector Machine.

### 3.5 Visualization of feature importance

**Figure 1:**
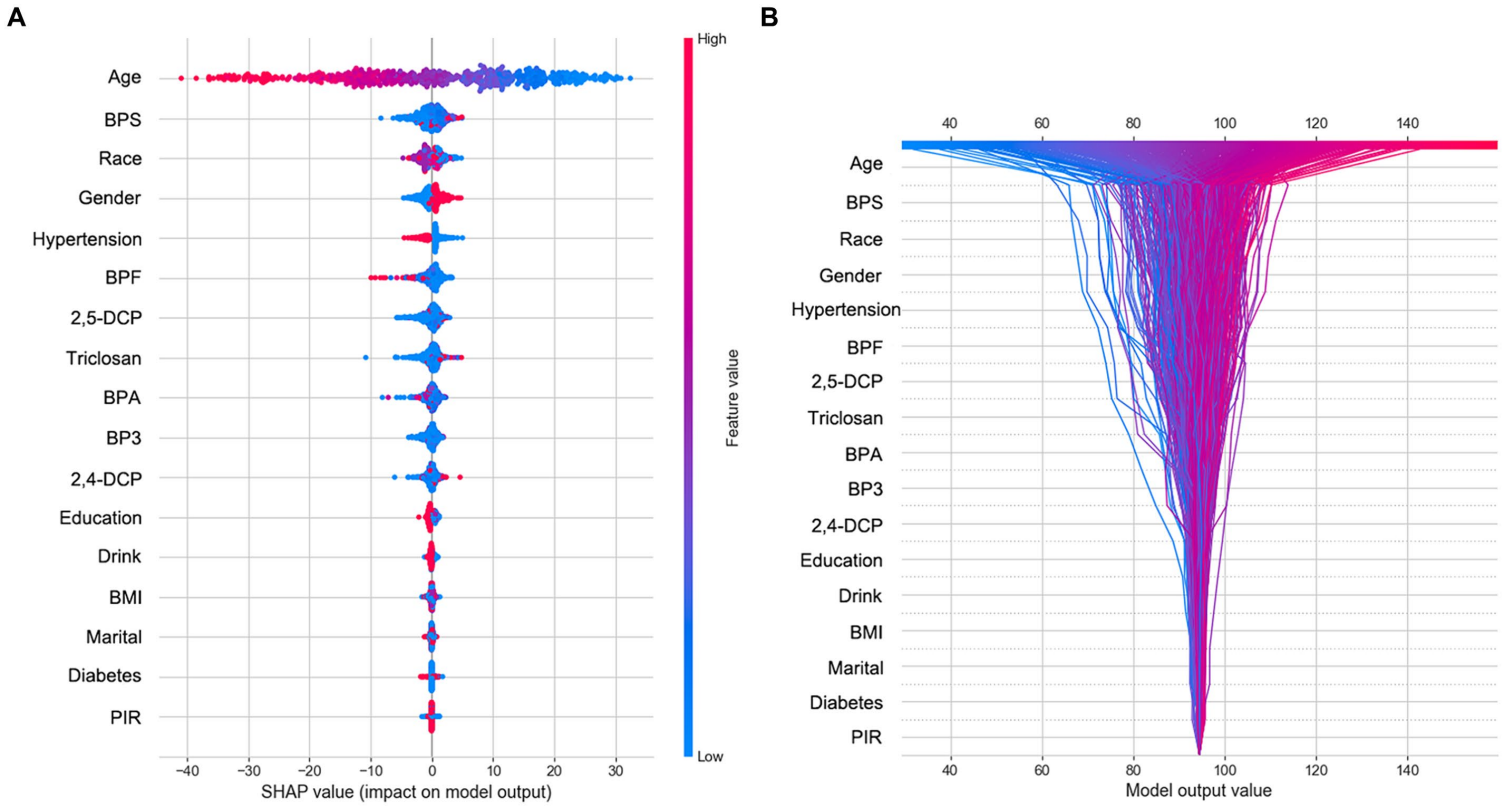
The SHAP summary and decision plot. **(A)** The dot SHAP value plot of participants’ features value effect on the eGFR. **(B)** The SHAP logic decision plot of participants’ features in person.

SHAP was utilized to graphically demonstrate the specified features’ impact on eGFR in the RF model. Figure 1A shows a graphical representation of specified features on eGFR in the RF model. This SHAP dot plot shows the influence of each variable in the ML model on predicting eGFR in the test datasets. SHAP value indicate Urinary Bisphenol S (1.38), Urinary Bisphenol *F* (0.97), 2,5-dichlorophenol (0.87), Urinary Triclosan (0.78), Urinary Benzophenone-3 (0.60), Urinary Bisphenol A (0.59) and 2,4-dichlorophenol (0.47) make negative contributions to the model. In addition, the plot shows old, hypertension, female are associated with negative effect of eGFR. Further, we applied SHAP interaction analysis to select 1,000 study participants randomly from the dataset. The bar chart on the Supplementary Figure S3 represents the influence of each feature on the RF model. It is the tremendous contribution of age effect on eGFR. The BPS is the most critical exposure in eGFR. We also transposed the matrix of SHAP values to the correlation plot with samples arranged based on hierarchical clustering by values; the correlation between BPF and age represents the whole age range effect on the eGFR; the BPS exposure has a negative effect on eGFR when age increase (Supplementary Figure S4).

### 3.6 Interpretation of personalized predictions

The decision plot in Figure 1B showcases each participant’s contribution to the outcome. Each line represents the predicted outcome value across the range of a specific feature, holding all other features constant. Age, a standout feature in our analysis, emerged as the most impactful. It demonstrates a clear positive association with the predicted outcome, suggesting that the model’s output value also tends to decrease as age increases. This underscores the significant role of age in our predictive model. Several other features, including BPS, Race, Gender, Hypertension, BPF, and 2,5-DCP, also exhibited notable influence, with varying degrees of positive and negative associations. This complexity in their influence adds depth to our analysis and underscores the need for a nuanced understanding of these features. The remaining features (Triclosan, BPA, BP3, 2,4-DCP, Education, Drink, BMI, Marital, Diabetes, and PIR) showed minimal individual impact on the predicted outcome, as the relatively flat ICE lines indicated. The computed features are arranged in descending order of importance over the plotted observations. All the lines converge at a common point, which is 94.375.

## 4 Discussion

In our research, we examined the use of a transparent ML technique in predicting eGFR based on environmental phenols’ exposure between 2013 and 2016, using data from the US NHANES. We evaluated five ML models and found that the RF model was the most effective, and therefore selected it for eGFR prediction. The RF model performed exceptionally well with an R score of 0.998, indicating high efficiency and stability for the regression model, and it showed a lower error rate of 0.68. We also employed the SHAP game theory approach to highlight each feature’s importance in the model, and the decision plot confirmed the RF model’s accuracy and robustness. In summary, our study suggests that the RF model, along with environmental phenols’ exposure data, has significant potential in predicting eGFR.

The NHANES, a cornerstone in US public health research, provides comprehensive and representative health and nutritional status data. It is imperative to consider both its methodological strengths and limitations. The survey’s comprehensive data collection strategy, combining interviews with physical examinations, offers a robust and detailed assessment of the health and nutritional status of the US population (29). This approach not only enhances the depth of the data but also contributes to its reliability and validity. Additionally, NHANES employs a sophisticated, multi-tiered probability sampling methodology, resulting in a sample that accurately reflects the broader US civilian non-institutionalized population. This approach enhances the applicability of its discoveries. Moreover, the survey’s structure supports longitudinal research, granting valuable perspectives into the ever-changing landscape of health and nutrition (30).

However, its effectiveness could be improved by high costs, potential biases, and time-consuming data collection and analysis processes (31). Integrating ML methodologies offers a transformative opportunity to address these challenges and enhance the utility of NHANES. ML algorithms’ ability to efficiently analyze large datasets can reveal intricate patterns and correlations, offering more profound insights into public health trends and facilitating more accurate predictive modeling. This enhancement is pivotal for refining public health strategies and ensuring timely responses to health crises. Simultaneously, the application of ML can mitigate NHANES’s logistical and financial constraints by automating data processing tasks, thus improving cost-effectiveness (32). ML’s proficiency in handling missing data also bolsters the dataset’s robustness, enhancing the survey’s reliability. Furthermore, ML can identify and correct sampling biases, ensuring broader applicability and representativeness of the findings (33).

Our methodology involved training and evaluating ML models using this extensive dataset, focusing particularly on assessing individual exposure to environmental phenols through urinary analysis. To enhance the robustness of our models against potential biases arising from temporal changes in phenols exposure levels, we excluded the average exposure data of study participants from the training dataset. This approach ensures that the models are not influenced by the downward trend in exposure following the legislative ban and increased public awareness (34, 35). Consequently, our models provide a more stable and reliable analysis of bisphenol exposure’s impact on health, unaffected by external temporal factors.

We employed five ML methods to predict eGFR based on environmental phenols exposure, which have been previously shown to be effective in predicting various diseases in other contemporary ML studies (36–38). Then, we conducted a comprehensive assessment of the predictive capabilities of the ML models, by utilizing test datasets to evaluate each model’s discrimination abilities. Our findings revealed that the R score of the RF model was 0.998, indicating good stability for our model for all range exposure level of environmental phenols. Other models, however, are less impressive. KNN suffers from high computational cost in large datasets as it requires computing and comparing distances for all data points during prediction (39). SVM perform poorly and are computationally intensive with large datasets or datasets with a high number of features, due to their reliance on solving quadratic programming problems (40). DT are prone to overfitting, especially with complex datasets, as they tend to learn too much from the training data, including its noise and outliers (41). AdaBoost can be sensitive to noisy data and outliers, as it tends to focus excessively on hard-to-classify instances, which can decrease its overall performance (42). At last, the RF model exhibited the highest classification robustness. Specifically, the discrimination characteristics provided a comprehensive indication of the ML models’ performance. We recognized the challenge of accurately comprehending the ML methodology and visually presenting the identity results in a practical way. We made the decision to incorporate SHAP values in combination with the RF model to achieve the most efficient means of assessing identity impact and improving interpretability. A negative SHAP value indicates that the feature’s associated values resulted in a lower eGFR value, while a positive SHAP value suggests a higher value. The SHAP by tree-regressor explainer is a helpful tool that assists individuals in visualizing the model’s regression process (43).

The results obtained by applying SHAP values are consistent with the results of earlier studies. A study using the Korean National Environmental Health Survey found that BPA and eGFR showed a significant negative correlation (44). A joint effect model based on quantile g calculations showed that quartile increases in EDC mixtures corresponded to decreases in eGFR, with BPA identified as the major contributor to this effect (45). However, some studies have reported a positive correlation between BPA and eGFR, which is contrary to the observations of this study (46). This may be because a decrease in eGFR is accompanied by a decrease in the excretion of chemicals in the urine, thus producing conflicting results (47, 48). For BPF, its impact on eGFR has not yet been found at the population level, but a result based on metabolomics and lipidomics shows: BPF exposure will disrupt the metabolome and lipid profile of the liver and kidneys, causing renal tissue membrane homeostasis and cell dysfunction by disrupting biosynthesis and glycolysis metabolism in liver and kidney tissues, thereby causing renal function damage (49). In addition, results from a rat experiment showed that genetic background modifies the effect of BPF exposure on kidney weight (50). When linear regression analysis was applied, we observed a positive correlation between BPS and TCS and eGFR, an observation that suggests these chemicals may have a protective effect. A study that also used NHANES data showed that the excretion of triclosan in urine decreased with the decline of renal function (48), which was similar to the observations of this study. In fact, TCS is a common broad-spectrum antibacterial agent that inhibits the growth of bacteria, fungi and some viruses by inhibiting bacterial fatty acid synthesis (51). This may be one of the reasons for its protective effect on the kidneys. Previous studies have found that exposure to BPS affects the oxidative stress, cell viability, apoptosis levels and catalase (CAT) activity of mouse kidney cells, thereby causing kidney damage (13, 52). However, this study focuses on the general population. The exposure level of BPS in urine is low, which may produce a toxic hormesis effect on the body (53). Therefore, the potential effects of BPS and TCS on renal function still require further study.

It’s crucial to recognize that our validation approach was limited by a cross-sectional design, preventing us from establishing a causal link between environmental exposure and eGFR decline. However, the potential of future studies utilizing longitudinal data and prospective designs to confirm the temporal relationship observed in this study is truly intriguing. Further validation of our findings could be robustly achieved through a prospective cohort study, incorporating repeated measurements of environmental phenol exposure and eGFR over an extended period. This comprehensive approach would provide a more robust assessment of the temporal relationship between exposure and kidney function decline. While our analysis diligently controlled for several potential confounders, it’s crucial to underscore the potential impact of unmeasured factors. For instance, dietary habits, a known contributor to both environmental phenol exposure and kidney health, were not fully captured in our study (54). The inclusion of detailed dietary assessments in future research could be instrumental in clarifying the independent role of environmental phenols, thereby enriching our understanding of their effects on kidney health. An alternative explanation could involve genetic factors influencing both individual susceptibility to phenol toxicity and predisposition to kidney disease. Future studies investigating gene–environment interactions, particularly those involving genes implicated in phenol metabolism and organ function, such as 16S rRNA, could provide valuable insights (55).

Since the United States Food and Drug Administration announced legislation to ban BPA in 2012, public health has attached great importance to the impact of BPA and its substitutes on the population. Initially, concerns grew over BPA’s estrogen-mimicking properties, which have been linked to a variety of health problems, including endocrine disruption and developmental issues. Therefore, BPS and BPF have become alternatives to BPA (56). However, recent scientific scrutiny reveals that BPS and BPF share a striking chemical similarity to BPA, casting doubt on their safety (57). Detailly, like BPA, both BPS and BPF exhibit estrogenic activity, potentially leading to similar adverse health effects (58). However, with the deepening of research, the complex nonlinear relationship between independent variables and outcomes has posed great challenges to the application of traditional linear statistical methods, and the impact of synergistic effects between independent variables is still a controversial issue. In this study, we use a SHAP game theory approach to highlight the importance of each selected feature in the model, using a combination of traditional statistics and machine learning. A better fitting effect was achieved, and the potential impact of environmental phenol exposure on eGFR was also explored. In the coming years, the introduction of machine learning algorithms in medicine will provide professionals with more comprehensive insights, allowing them to make more informed decisions.

## 5 Conclusion

In the research conducted, the RF algorithm demonstrated effectiveness, precision, and resilience when exploring the links between phenols exposure and estimated eGFR in participants of the United States NHANES from 2013 to 2016. The findings indicate that BPA, BPF, and BPS are inversely associated with eGFR.

## Data availability statement

The original contributions presented in the study are included in the article/Supplementary material, further inquiries can be directed to the corresponding author/s.

## Ethics statement

The studies involving humans were approved by Research Ethics Review Board (ERB) of the United States National Center for Healthcare Statistics (NCHS) authorized the 2013–2016 NHANES. The studies were conducted in accordance with the local legislation and institutional requirements. The participants provided their written informed consent to participate in this study.

## Author contributions

LL: Conceptualization, Funding acquisition, Investigation, Writing – original draft. HZ: Conceptualization, Writing – original draft. XW: Formal analysis, Visualization, Writing – original draft. FW: Formal analysis, Visualization, Writing – original draft. GZ: Formal analysis, Visualization, Writing – original draft. JY: Formal analysis, Visualization, Writing – original draft. HS: Formal analysis, Visualization, Writing – original draft. RH: Funding acquisition, Project administration, Writing – review & editing.

## References

[1] Collaboration GBDCKD. Global, regional, and national burden of chronic kidney disease, 1990-2017: a systematic analysis for the global burden of disease study 2017. Lancet. (2020) 395:709–33. doi: 10.1016/S0140-6736(20)30045-3, PMID: 32061315 PMC7049905

[2] Levey AS Coresh J Tighiouart H Greene T Inker LA. Measured and estimated glomerular filtration rate: current status and future directions. Nat Rev Nephrol. (2020) 16:51–64. doi: 10.1038/s41581-019-0191-y, PMID: 31527790

[3] Stevens LA Coresh J Greene T Levey AS. Assessing kidney function--measured and estimated glomerular filtration rate. N Engl J Med. (2006) 354:2473–83. doi: 10.1056/NEJMra054415 16760447

[4] Mullens W Damman K Testani JM Martens P Mueller C Lassus J. Evaluation of kidney function throughout the heart failure trajectory - a position statement from the heart failure Association of the European Society of cardiology. Eur J Heart Fail. (2020) 22:584–603. doi: 10.1002/ejhf.1697, PMID: 31908120

[5] Matsushita K Coresh J Sang Y Chalmers J Fox C Guallar E. Estimated glomerular filtration rate and albuminuria for prediction of cardiovascular outcomes: a collaborative meta-analysis of individual participant data. Lancet Diabetes Endocrinol. (2015) 3:514–25. doi: 10.1016/S2213-8587(15)00040-6, PMID: 26028594 PMC4594193

[6] Ford ES. Urinary albumin-creatinine ratio, estimated glomerular filtration rate, and all-cause mortality among US adults with obstructive lung function. Chest. (2015) 147:56–67. doi: 10.1378/chest.13-2482, PMID: 25079336 PMC4580968

[7] Jha V Garcia-Garcia G Iseki K Li Z Naicker S Plattner B. Chronic kidney disease: global dimension and perspectives. Lancet. (2013) 382:260–72. doi: 10.1016/S0140-6736(13)60687-X, PMID: 23727169

[8] Liang Y Zhou H Zhang J Li S Shen W Lei L. Exposure to perfluoroalkyl and polyfluoroalkyl substances and estimated glomerular filtration rate in adults: a cross-sectional study based on NHANES (2017-2018). Environ Sci Pollut Res Int. (2023) 30:57931–44. doi: 10.1007/s11356-023-26384-9, PMID: 36971931

[9] Cao H Wang F Liang Y Wang H Zhang A Song M. Experimental and computational insights on the recognition mechanism between the estrogen receptor alpha with bisphenol compounds. Arch Toxicol. (2017c) 91:3897–912. doi: 10.1007/s00204-017-2011-0, PMID: 28616630

[10] Abraham A Chakraborty P. A review on sources and health impacts of bisphenol a. Rev Environ Health. (2020) 35:201–10. doi: 10.1515/reveh-2019-0034, PMID: 31743105

[11] Gramec Skledar D Peterlin ML. Bisphenol a and its analogs: do their metabolites have endocrine activity? Environ Toxicol Pharmacol. (2016) 47:182–99. doi: 10.1016/j.etap.2016.09.014, PMID: 27771500

[12] Priego AR Parra EG Mas S Morgado-Pascual JL Ruiz-Ortega M Rayego-Mateos S. Bisphenol a modulates autophagy and exacerbates chronic kidney damage in mice. Int J Mol Sci. (2021) 22:189. doi: 10.3390/ijms22137189, PMID: 34281243 PMC8268806

[13] Mandrah K Jain V Shukla S Ansari JA Jagdale P Ayanur A. A study on bisphenol S induced nephrotoxicity and assessment of altered downstream kidney metabolites using gas chromatography-mass spectrometry based metabolomics. Environ Toxicol Pharmacol. (2022) 93:103883. doi: 10.1016/j.etap.2022.103883, PMID: 35550874

[14] Kang H Lee JP Choi K. Exposure to phthalates and environmental phenols in association with chronic kidney disease (CKD) among the general US population participating in multi-cycle NHANES (2005-2016). Sci Total Environ. (2021) 791:148343. doi: 10.1016/j.scitotenv.2021.148343, PMID: 34126474

[15] Moor M Banerjee O Abad ZSH Krumholz HM Leskovec J Topol EJ. Foundation models for generalist medical artificial intelligence. Nature. (2023) 616:259–65. doi: 10.1038/s41586-023-05881-4, PMID: 37045921

[16] Segar MW Hall JL Jhund PS Powell-Wiley TM Morris AA Kao D. Machine learning-based models incorporating social determinants of health vs traditional models for predicting in-hospital mortality in patients with heart failure. JAMA Cardiol. (2022) 7:844–54. doi: 10.1001/jamacardio.2022.1900, PMID: 35793094 PMC9260645

[17] Statistics. NCfH, Prevention. CfDCa. *National Center for Health Statistics: National Health and nutrition examination survey (NHANES). Centers for Disease Control and Prevention*. Statistics. NCfH, Prevention. CfDCa (2024). Available at: https://www.cdc.gov/nchs/nhanes/index.htm.

[18] Khalid UB Haroon ZH Aamir M Ain QU Mansoor K Jaffar SR. Comparison of estimated glomerular filtration rate with both serum creatinine and cystatin C (eGFRcr-cys) versus single Analyte (eGFRcr or eGFRcys) using CKD-EPI and MDRD equations in tertiary care hospital settings. J Coll Physicians Surg Pak. (2020) 30:701–6. doi: 10.29271/jcpsp.2020.07.701, PMID: 32811598

[19] Levey AS Stevens LA Schmid CH Zhang YL Castro AF Feldman HI. A new equation to estimate glomerular filtration rate. Ann Intern Med. (2009) 150:604–12. doi: 10.7326/0003-4819-150-9-200905050-00006 19414839 PMC2763564

[20] Hao L Huang G. An improved AdaBoost algorithm for identification of lung cancer based on electronic nose. Heliyon. (2023) 9:e13633. doi: 10.1016/j.heliyon.2023.e13633, PMID: 36915521 PMC10006450

[21] Wang H Shao Y Zhou S Zhang C Xiu N. Support vector machine classifier via L (0/1) soft-margin loss. IEEE Trans Pattern Anal Mach Intell. (2022) 44:7253–65. doi: 10.1109/TPAMI.2021.3092177, PMID: 34166185

[22] Huang R Cai L Ma X Shen K. Autophagy-mediated circ HIPK2 promotes lipopolysaccharide-induced astrocytic inflammation via SIGMAR1. Int Immunopharmacol. (2023) 117:109907. doi: 10.1016/j.intimp.2023.109907, PMID: 36827915

[23] Che D Liu Q Rasheed K Tao X. Decision tree and ensemble learning algorithms with their applications in bioinformatics. Adv Exp Med Biol. (2011) 696:191–9. doi: 10.1007/978-1-4419-7046-6_19, PMID: 21431559

[24] Wang J Geng X. Large margin weighted k-nearest neighbors label distribution learning for classification. IEEE Trans Neural Netw Learn Syst. (2023) 2023:1–13. doi: 10.1109/TNNLS.2023.3297261 37527329

[25] Methavigul K Sairat P Krittayaphong R Investigators C-A. Efficacy of R (2)CHA(2)DS(2)-VA score for predicting thromboembolism in Thai patients with non-valvular atrial fibrillation. BMC Cardiovasc Disord. (2021) 21:540. doi: 10.1186/s12872-021-02370-2, PMID: 34772351 PMC8588707

[26] Trimboli RM Codari M Cozzi A Monti CB Capra D Nenna C. Semiquantitative score of breast arterial calcifications on mammography (BAC-SS): intra-and inter-reader reproducibility. Quant Imaging Med Surg. (2021) 11:2019–27. doi: 10.21037/qims-20-560, PMID: 33936983 PMC8047367

[27] Tseng PY Chen YT Wang CH Chiu KM Peng YS Hsu SP. Prediction of the development of acute kidney injury following cardiac surgery by machine learning. Crit Care. (2020) 24:478. doi: 10.1186/s13054-020-03179-9, PMID: 32736589 PMC7395374

[28] Fan Z Jiang J Xiao C Chen Y Xia Q Wang J. Construction and validation of prognostic models in critically ill patients with sepsis-associated acute kidney injury: interpretable machine learning approach. J Transl Med. (2023) 21:406. doi: 10.1186/s12967-023-04205-4, PMID: 37349774 PMC10286378

[29] Liu Z Kuo PL Horvath S Crimmins E Ferrucci L Levine M. A new aging measure captures morbidity and mortality risk across diverse subpopulations from NHANES IV: a cohort study. PLoS Med. (2018) 15:e1002718. doi: 10.1371/journal.pmed.1002718, PMID: 30596641 PMC6312200

[30] Iranpour S Sabour S. Inverse association between caffeine intake and depressive symptoms in US adults: data from National Health and nutrition examination survey (NHANES) 2005-2006. Psychiatry Res. (2019) 271:732–9. doi: 10.1016/j.psychres.2018.11.004, PMID: 30791349

[31] Xing W Gao W Zhao Z Xu X Bu H Su H. Dietary flavonoids intake contributes to delay biological aging process: analysis from NHANES dataset. J Transl Med. (2023) 21:492. doi: 10.1186/s12967-023-04321-1, PMID: 37480074 PMC10362762

[32] Cheng TD Ferderber C Kinder B Wei YJ. Trends in dietary vitamin a intake among US adults by race and ethnicity, 2003-2018. JAMA. (2023) 329:1026–9. doi: 10.1001/jama.2023.0636, PMID: 36976287 PMC10051065

[33] Liu S Ben X Liang H Fei Q Guo X Weng X. Association of acrylamide hemoglobin biomarkers with chronic obstructive pulmonary disease in the general population in the US: NHANES 2013-2016. Food Funct. (2021) 12:12765–73. doi: 10.1039/d1fo02612g, PMID: 34851334

[34] Wei H Sun J Shan W Xiao W Wang B Ma X. Environmental chemical exposure dynamics and machine learning-based prediction of diabetes mellitus. Sci Total Environ. (2022) 806:150674. doi: 10.1016/j.scitotenv.2021.150674, PMID: 34597539

[35] Klados GA Politof K Bei ES Moirogiorgou K Anousakis-Vlachochristou N Matsopoulos GK. Machine learning model for predicting CVD risk on NHANES data. Annu Int Conf IEEE Eng Med Biol Soc. (2021) 2021:1749–52. doi: 10.1109/EMBC46164.2021.9630119, PMID: 34891625

[36] Dong J Feng T Thapa-Chhetry B Cho BG Shum T Inwald DP. Machine learning model for early prediction of acute kidney injury (AKI) in pediatric critical care. Crit Care. (2021) 25:288. doi: 10.1186/s13054-021-03724-0, PMID: 34376222 PMC8353807

[37] Sealfon RSG Mariani LH Kretzler M Troyanskaya OG. Machine learning, the kidney, and genotype-phenotype analysis. Kidney Int. (2020) 97:1141–9. doi: 10.1016/j.kint.2020.02.028, PMID: 32359808 PMC8048707

[38] Ye Z An S Gao Y Xie E Zhao X Guo Z. The prediction of in-hospital mortality in chronic kidney disease patients with coronary artery disease using machine learning models. Eur J Med Res. (2023) 28:33. doi: 10.1186/s40001-023-00995-x, PMID: 36653875 PMC9847092

[39] Zhang S Li X Zong M Zhu X Wang R. Efficient kNN classification with different numbers of nearest neighbors. IEEE Trans Neural Netw Learn Syst. (2018) 29:1774–85. doi: 10.1109/TNNLS.2017.2673241, PMID: 28422666

[40] Reynolds E Callaghan B Banerjee M. SVM-CART for disease classification. J Appl Stat. (2019) 46:2987–3007. doi: 10.1080/02664763.2019.1625876, PMID: 33012942 PMC7531767

[41] Schwebel FJ Richards DK Pfund RA Joseph VW Pearson MR Marijuana Outcomes Study T. Using decision trees to identify salient predictors of Cannabis-related outcomes. J Psychoactive Drugs. (2022) 54:419–28. doi: 10.1080/02791072.2021.2014081, PMID: 35067209 PMC9308832

[42] Tang J Henderson A Gardner P. Exploring AdaBoost and random forests machine learning approaches for infrared pathology on unbalanced data sets. Analyst. (2021) 146:5880–91. doi: 10.1039/d0an02155e, PMID: 34570844

[43] Wang Y Lang J Zuo JZ Dong Y Hu Z Xu X. The radiomic-clinical model using the SHAP method for assessing the treatment response of whole-brain radiotherapy: a multicentric study. Eur Radiol. (2022) 32:8737–47. doi: 10.1007/s00330-022-08887-0, PMID: 35678859

[44] Lee I Park JY Kim S An JN Lee J Park H. Association of exposure to phthalates and environmental phenolics with markers of kidney function: Korean National Environmental Health Survey (KoNEHS) 2015-2017. Environ Int. (2020) 143:105877. doi: 10.1016/j.envint.2020.105877 32645486

[45] Chen Q Deng Q Liao Q Liu Y Zhang Z Wu D. 8-OHdG mediates the association of co-exposure to fifty-five typical endocrine-disrupting chemicals with renal function: a cross-section investigation in southern Chinese adults. Environ Sci Pollut Res Int. (2024) 31:30779–92. doi: 10.1007/s11356-024-33266-1, PMID: 38613763

[46] Malits J Attina TM Karthikraj R Kannan K Naidu M Furth S. Renal function and exposure to bisphenol a and phthalates in children with chronic kidney disease. Environ Res. (2018) 167:575–82. Disclosure: The authors have no financial conflicts of interests to declare. Disclosure statement: There are no conflicts of interest to report. doi: 10.1016/j.envres.2018.08.006, PMID: 30172191 PMC7409562

[47] Jin R Zhu X Shrubsole MJ Yu C Xia Z Dai Q. Associations of renal function with urinary excretion of metals: evidence from NHANES 2003-2012. Environ Int. (2018) 121:1355–62. Epub 20181112. doi: 10.1016/j.envint.2018.11.002, PMID: 30442456

[48] You L Zhu X Shrubsole MJ Fan H Chen J Dong J. Renal function, bisphenol a, and alkylphenols: results from the National Health and nutrition examination survey (NHANES 2003-2006). Environ Health Perspect. (2011) 119:527–33. doi: 10.1289/ehp.1002572, PMID: 21147601 PMC3080936

[49] Zhao C Xie P Wang H Cai Z. Liquid chromatography-mass spectrometry-based metabolomics and lipidomics reveal toxicological mechanisms of bisphenol F in breast cancer xenografts. J Hazard Mater. (2018) 358:503–7. doi: 10.1016/j.jhazmat.2018.05.010, PMID: 29759594

[50] Wagner VA Holl KL Clark KC Reho JJ Dwinell MR Lehmler HJ. Genetic background in the rat affects endocrine and metabolic outcomes of bisphenol F exposure. Toxicol Sci. (2023) 194:84–100. doi: 10.1093/toxsci/kfad046, PMID: 37191987 PMC10306406

[51] Shrestha P Zhang Y Chen WJ Wong TY. Triclosan: antimicrobial mechanisms, antibiotics interactions, clinical applications, and human health. J Environ Sci Health C Toxicol Carcinog. (2020) 38:245–68. doi: 10.1080/26896583.2020.1809286, PMID: 32955413

[52] Zhang R Liu R Zong W. Bisphenol S interacts with catalase and induces oxidative stress in mouse liver and renal cells. J Agric Food Chem. (2016) 64:6630–40. doi: 10.1021/acs.jafc.6b02656, PMID: 27508457

[53] Duan L Chen Q Duan S. Transcriptional analysis of *Chlorella pyrenoidosa* exposed to bisphenol a. Int J Environ Res Public Health. (2019) 16:1374. doi: 10.3390/ijerph16081374 30995802 PMC6518184

[54] Li N Li S Wang Y Wang Q Zhou J Liu J. Compositional analysis and immunomodulatory activity of blue pigment fraction (BPF) from Laba garlic. Food Chem. (2023) 406:134976. doi: 10.1016/j.foodchem.2022.134976 36455311

[55] Gu J Zhu Y Guo M Yin X Liang M Lou X. The potential mechanism of BPF-induced neurotoxicity in adult zebrafish: correlation between untargeted metabolomics and gut microbiota. Sci Total Environ. (2022) 839:156221. doi: 10.1016/j.scitotenv.2022.156221, PMID: 35623532

[56] Wu LH Zhang XM Wang F Gao CJ Chen D Palumbo JR. Occurrence of bisphenol S in the environment and implications for human exposure: a short review. Sci Total Environ. (2018) 615:87–98. doi: 10.1016/j.scitotenv.2017.09.194 28963899

[57] Thoene M Dzika E Gonkowski S Wojtkiewicz J. Bisphenol S in food causes hormonal and obesogenic effects comparable to or worse than bisphenol a: a literature review. Nutrients. (2020) 12:532. doi: 10.3390/nu12020532, PMID: 32092919 PMC7071457

[58] Kim JI Lee YA Shin CH Hong YC Kim BN Lim YH. Association of bisphenol a, bisphenol F, and bisphenol S with ADHD symptoms in children. Environ Int. (2022) 161:107093. doi: 10.1016/j.envint.2022.107093, PMID: 35077929

