# Supplementary material for: Effects of environmental phenols on eGFR: machine learning modeling methods applied to cross-sectional studies
Source: Front Public Health. 2024 Aug 1;12:1405533. doi: 10.3389/fpubh.2024.1405533 (PMC11324456; doi:10.3389/fpubh.2024.1405533)
Supplement: Supplementary file 1 [file Data_Sheet_1.docx]

Supplementary Material

# Supplementary Figures


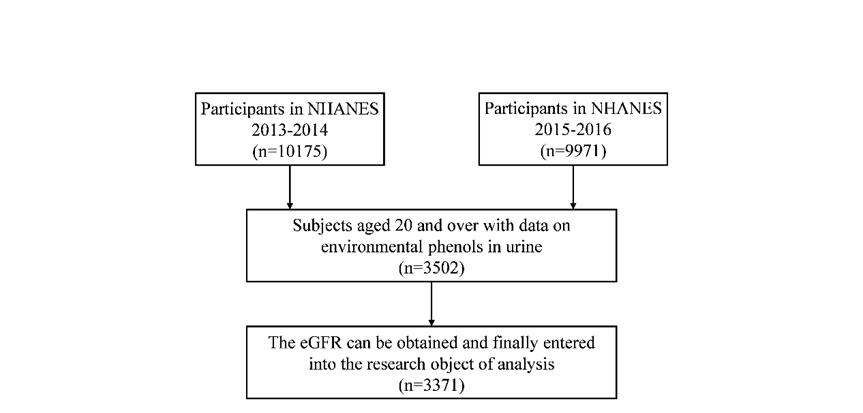


**Supplementary Figure 1.** Flowchart for selecting eligible participants from NHANES.

**
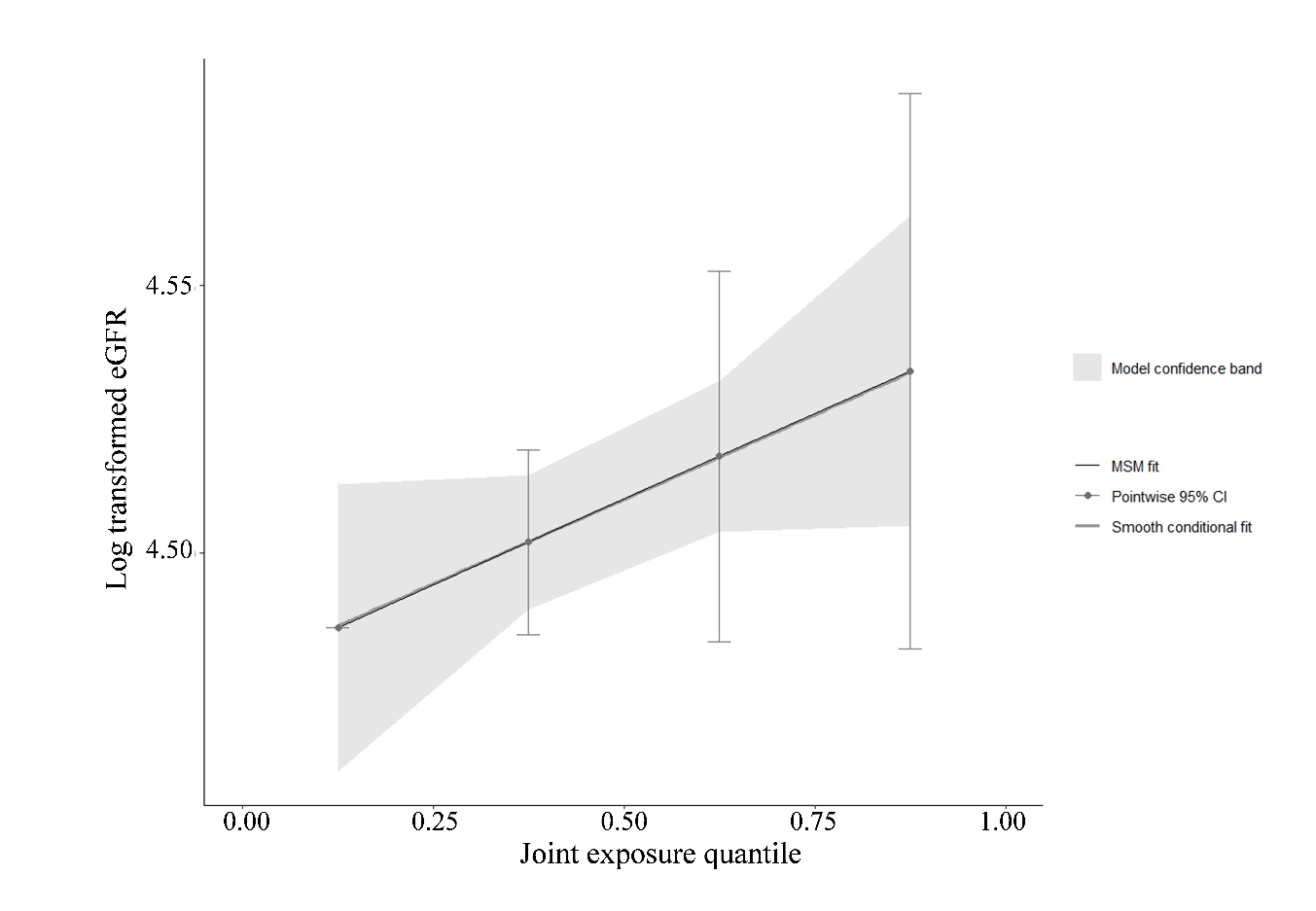
**

**Supplementary Figure 2.** Joint effect of environmental phenols on eGFR based on quantile g calculation (QGC) regression. Model adjusted for gender, age, race/ethnicity, poverty income ratio, body mass index, hypertension, and diabetes.


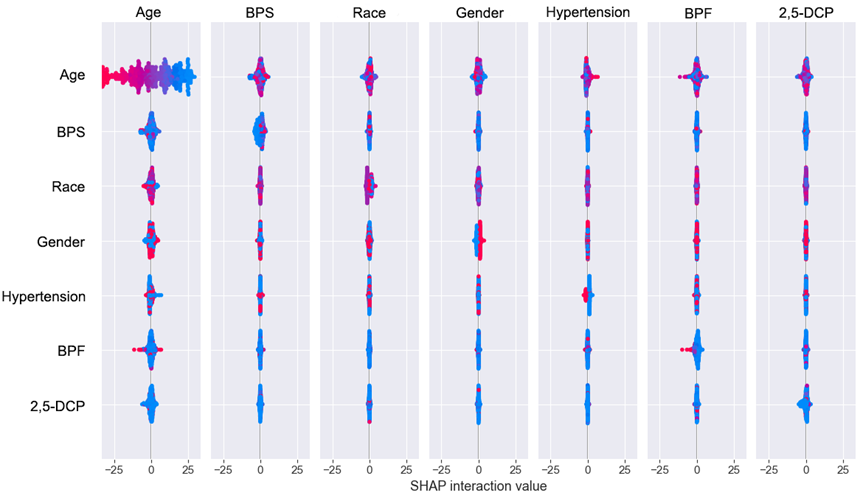


**Supplementary Figure 3.** The interaction SHAP value of feature on eGFR.


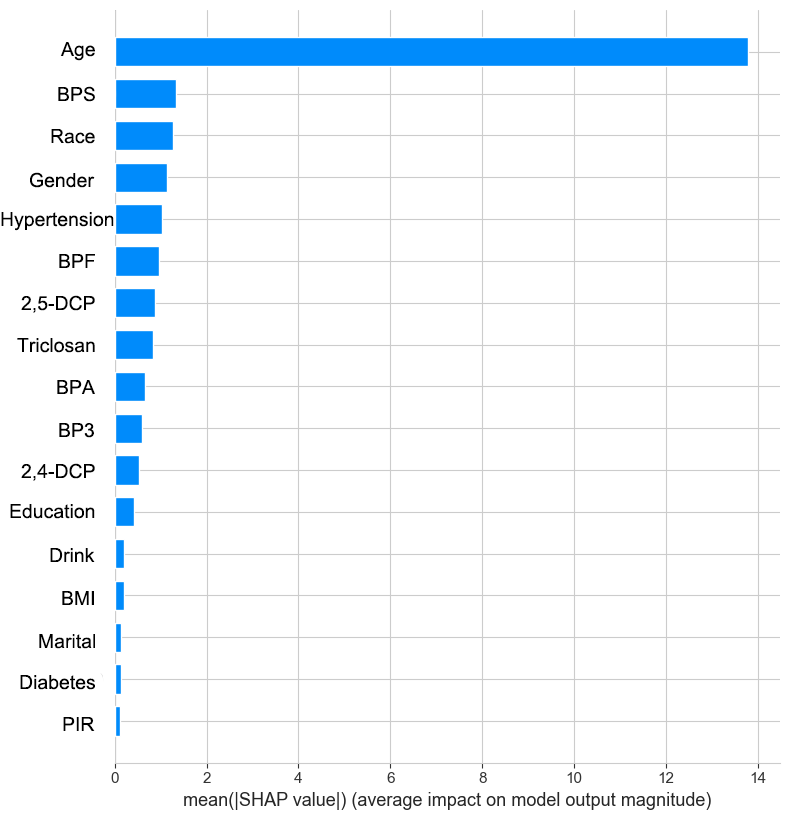


**Supplementary Figure 4.** The mean SHAP value of each feature on eGFR.

| Table S1. Association of urinary environmental phenols with eGFR (mL/min/1.73 m^2^) in regression model (n = 3371). | | |
| --- | --- | --- |
| Categories | *β* (95%CI) | *P*-Value |
| BPA | -0.006 (-0.014, 0.003) | 0.191 |
| BPS | 0.010 (0.001, 0.018) | 0.026 |
| BPF | -0.005 (-0.015, 0.005) | 0.341 |
| BP3 | 0.005 (-0.001, 0.010) | 0.054 |
| TCS | 0.007 (0.003, 0.012) | 0.004 |
| 2,5-DCP | 0.001 (-0.005, 0.006) | 0.847 |
| 2,4-DCP | 0.006 (-0.002, 0.014) | 0.117 |
| Note: The association was adjusted for gender, age, race/ethnicity, poverty: income ratio, educational level, marital status, body mass index, drinking, hypertension and diabetes. Creatinine-corrected urinary ambient phenols and eGFR were log-transformed. | | |
